# Fertility Does Not Alter Disease Progression in ALS Patients of Childbearing Age: A Three Centers Retrospective Analysis in Southern China

**DOI:** 10.3389/fneur.2022.895321

**Published:** 2022-06-30

**Authors:** Biying Yang, Sen Huang, Yu Zheng, Xiaomei Hou, Jianing Lin, Yu Peng, Baoxin Du, Xiaoli Yao

**Affiliations:** ^1^Department of Neurology, Guangdong Provincial Hospital of Chinese Medicine, The Second Affiliated Hospital of Guangzhou University of Chinese Medicine, Guangzhou, China; ^2^Department of Neurology, The First Affiliated Hospital, Sun Yat-sen University, Guangdong Provincial Key Laboratory of Diagnosis and Treatment of Major Neurological Diseases, National Key Clinical Department and Key Discipline of Neurology, Guangzhou, China; ^3^Department of Neurology, Nanfang Hospital, Southern Medical University, Guangzhou, China

**Keywords:** amyotrophic lateral sclerosis, pregnancy, fertility, disease progression, genetic

## Abstract

**Background:**

Limited data exists on the clinical features of patients with amyotrophic lateral sclerosis (ALS) during reproductive ages.

**Objective:**

Our study characterized the clinical features of ALS and the effects of pregnancy on disease progression in patients with ALS.

**Methods:**

We performed a retrospective study of female patients with ALS in three ALS research centers in southern China from 2009 to 2021. Data regarding fertility status, and clinical and genetic features, were collected. Age-matched male patients with ALS served as controls. The patients were divided into the following two subgroups: patients with symptom onset within 1 year of pregnancy and patients with symptom onset over 1 year group after pregnancy.

**Results:**

A total of 52 female and 52 matched male patients were enrolled. There were no differences in female and male patients in the mean age of symptom onset, the mean baseline ALSFRS-R score, or median reduction of ALSFRS-R score (*p* > 0.05). The mean age of first pregnancy was 25.57 ± 4.40) years. The mean age of first pregnancy in the over 1 year group was lower than that in the within 1 year group (*p*= 0.01). There was no difference in the median reduction of ALSFRS-R between the two subgroups. In the univariate analysis, diagnostic delay was highly correlated with the disease progression, with short delay representing rapid progress. No multicollinearity was found among every variable. In addition, 40.38% patients carried ALS-related gene variants. The proportion with gene mutations in the within 1 year group was higher than that in the over 1 year group (*p* < 0.01). Furthermore, SETX was the most frequently mutated gene in this cohort (16.67%) including 4 uncertain mutation.

**Conclusion:**

Pregnancy and fertility were not associated with disease progression. Diagnostic delay was correlated with disease progression in this cohort. In addition, SETX might be a gene of concern for ALS patients of childbearing age.

## Introduction

Amyotrophic lateral sclerosis (ALS) is a fatal neurodegenerative disorder characterized by progressive muscle weakness and muscular atrophy due to loss of upper and lower motor neurons (1). Recent studies have reported that the average age of onset in female patients was 53.34 (SD = 13.55) in China (2), which is younger than that observed in other countries (3–6). Implementation of the three-child policy in China has led to an increase in the population of women of advanced maternal age (35 years or older). Therefore, the proportion of patients with ALS in China in their reproductive years (15–49 years) has increased. Furthermore, the proportion of women diagnosed with ALS in close temporal proximity to their pregnancy, which in our study was defined as within 1 year (365 days) before or after the pregnancy (1 year before pregnancy was defined as the period between 395 and 30 days before conception) has also increased. However, whether pregnancy and fertility contribute to disease progression is unclear (7–10). Some studies have indicated that pregnancy could attenuate protection through elevated levels of estrogen and progesterone, which are associated with neuroprotection. In contrast, some studies have argued that pregnancy and delivery promoted progression of ALS due to decreased estrogen, neuro-inflammation, and cytokine activation. Most previous studies are case reports with an associated literature review. Therefore, we performed a descriptive study to further enrich this field of research.

Although the vast majority of ALS is sporadic (without a family history), 5%-10% is familial (11). ALS is recognized as a multifactorial heterogeneous disease with both genetic and environmental causes (12), and gene mutation has been assumed to play a critical role in early onset ALS (13). Superoxide dismutase 1 (SOD1) mutation was the most common genetic form in China (14, 15), followed by TARDBP and FUS. Mutations of C9orf72 were rare in China compared to those in Europe and North America (16, 17). The common types and frequency of gene mutations in patients of reproductive age with ALS have not been reported.

We investigated the fertility status, and clinical and genetic features of patients of reproductive age with ALS in three research centers in southern China. The primary aim was to determine whether pregnancy and fertility affected disease progression.

## Methods

### Study Design

We conducted a retrospective case-control study on male and female patients with ALS aged 15–49 years. Relevant clinical data including age, onset age, initial segment, disease duration, family history, and medicine history were collected. Disease progression was assessed using the mean of monthly decline of ALSFRS-R scores [Δ ALSFRS-R = (initial ALSFRS-R scores—follow-up ALSFRS-R scores) / follow-up time (months)]. The ALSFRS-R scores were collected through telephone interviews or outpatient follow-up visits once within 6–12 months. Then, the patients were divided into the following two subgroups: onset of ALS within 1 year of pregnancy and onset of ALS over 1 year after pregnancy to compare their clinical features. In order to exclude the influence of confounding factors affecting disease progression, univariate and multivariate linear regression were carried out.

### Participants

Data from female patients with ALS were collected from the electronic case record of three research centers of ALS in southern China, including the First Affiliated Hospital of Sun Yat-Sen University, the Nanfang Hospital, Southern Medical University and the Guangdong Provincial Hospital of Chinese Medicine from March 2009 to June 2021. All patients were diagnosed with ALS/probable ALS according to El Escorial criteria (1994) by at least two neurologists. Reproductive age was defined as 15–49 years old with regular menstrual cycles. Women who underwent ovariectomy, hysterectomy, or who had used oral contraceptives or hormone replacement therapy were excluded. Male controls were recruited through the same three centers with a 1:1 match and were diagnosed according to the same criteria and matched for age (± 3 years), with the exception of spouses or blood-relatives of the patient to prevent overmatching. Written informed consent was obtained from all participants. This study was approved by Ethic Committee for clinical research and animal trials of the three research centers. The study approval numbers were H2015-012-02, NFEC-2017-029, and 2021260.

### Genetic Tests

Genomic DNA was extracted using a QIAamp Blood Midi Kit (QIAGEN, Valencia, CA). To identify disease-causing gene variants, a GenCap panel with 84 genes (see Supplementary Table 1) associated with ALS was customized, and a capture strategy was performed using the GenCap custom enrichment kit (MyGenostics Inc, Beijing, China). An Illumina NextSeq 500 sequencer (Illumina, San Diego, CA, United States) was used with 150 bp paired-end reads. An ABI3730xl sequencer (Applied Biosystems, United States) was used to apply the Sanger sequencing method, and the results were compared to the capture sequencing results (18, 19).

After sequencing, the raw data were saved in FASTQ format. Quality control (QC) filters were applied to remove reads with low quality (Q30>90%). Then, the clean reads were assembled and spliced using the second-generation sequencing analysis platform provided by MyGenostics and the coverage and sequencing quality of the target region were evaluated. Finally, flash analysis platform was used to analyze the pathogenicity of variation, and the possible variation loci were determined. The pathogenicity of variation loci was also analyzed according to ACMG (American College of Medical Genetics and Genomics) genetic variation classification criteria and guidelines. The target sequences covering the (G4C2) n tracts in the C9ORF72 gene, which could not be detected by exome capture analysis, were analyzed using repeat-primed PCR.

### Statistical Analysis

Statistical analysis was performed using SPSS 22.0 (IBM, Armonk, NY) software. Continuous data with a normal distribution were presented as the mean ± standard deviation. Continuous variables with non-normal distributions were presented as the median and range. Categorical variables were presented as proportions. Student's *t*-test was used to compare the onset ages and baseline ALSFRS-R scores between the two groups. Non–parametric tests were used to evaluate the mean diagnostic delay, the age of first pregnancy, and the median reduction of ALSFRS-R. Chi-squared tests were used to analyze site of onset, use of medication, number of pregnancies and live births, and the proportion of gene mutations. Univariate and multivariate linear regression were carried out to calculate the odds ratios (OR) for the risk of high progression of ALS. Variables were included in the multivariate analysis with enter approach. The variables selected by the model included age at onset, age at first pregnancy, site of onset, medication use, diagnostic delay and previous pregnancy. Tolerance <0.1 or variance inflation factor (VIF) >10 was considered to exist the multicollinearity in the model. *P* < 0.05 was regarded as statistically significant.

## Results

### Characteristics of the Male and Female Patients Affected by ALS Aged 15–49 Years

In our study, 52 female and 52 male patients with ALS were recruited. The clinical data are summarized in Table 1. The mean age of symptom onset was 37.81 years (± 6.83), with a median age of 38.00 years in female patients. The mean diagnostic delay was 12 (3–48) months. The mean ALSFRS-R score of female patients at baseline was 37.65 (± 5.63; range 24–46). The percentage of patients exhibiting spinal onset was higher than that of patients with bulbar onset (41/52 spinal vs. 11/52 bulbar). In addition, 88.46% of the subjects had received riluzole or Edaravone at some point. Compared with female patients, there was no difference in the mean age of symptom onset (38.00± 6.38), site of onset (48/52 spinal vs. 4/52 bulbar), diagnostic delay (the median time 11 months) or the mean ALSFRS-R total score 39.25 (± 5.06) in male patients. The median reduction of ALSFRS-R (Δ ALSFRS-R) was 0.65 (0.11, 6.00) in female patients and 0.75 (0.08, 2.86) in male patients respectively, which indicated a faster decline in men than in women, but the difference was not statistically significant (*p* = 0.83) (Table 1). Three women who had never been pregnant were excluded, but exclusion of these patients did not alter the Δ ALSFR-R results between the two groups (*p* = 0.49).

**Table 1:** Clinical characteristics of male and female patients affected by ALS aged 15–49 years.

	**Women** **(*N* = 52)**	**Men** **(*N* = 52)**	***p*** **-value**
Age of onset (years)	37.81 (6.83)	38.00 (6.38)	0.87
Median [Min, Max]	38.00 (22.00,49.00)	38.00 (23.00,49.00)	
Diagnostic delay (months) Median (Min, Max)	12 (3,48)	11 (2, 60)	0.14
Site of onset			
Bulbar	11 (21.15%)	4 (7.7%)	0.05
Spinal	41 (78.85%)	48 (92.31%)	
ALSFRS-R total score at baseline	37.65 ± 5.63	39.25± 5.06	0.13
Δ ALSFRS-R	0.65 (0.11,6.00)	0.75 (0.08,2.86)	0.83
Medication use			
Yes	46 (88.46%)	48 92.31%)	0.51
No	6 (11.54%)	4 (7.69%)	

### Comparison of Clinical Characteristics Between Patients With ALS Who Experienced Pregnancy Within 1 Year of Diagnosis and More Than 1 Year From Diagnosis

To determine whether pregnancy or childbirth affected ALS progression, we divided the female patients into two groups. There were nine patients (17.31%) diagnosed within 1 year before or after pregnancy (Group Within 1 year). The remaining 40 patients (76.92%) were diagnosed more than 1 year after childbirth, and three patients (5.77%) were never pregnant (Group Over 1 year). The average onset age in the within 1 year group was younger than that in the over 1 year group (*p* < 0.05). There were no differences in the mean ALSFRS-R scores at baseline, site of onset, diagnostic delay, or medication use between the two groups. Still, Δ ALSFRS-R did not show any difference between the two subgroups (Table 2).

**Table 2:** Comparison of the clinical characteristics between patients with ALS within 1 year and over 1 year between pregnancy and symptom onset.

	**Within 1 year** **(*N* = 9)**	**Over 1 year** **(*N* = 43)**	***p*** **-value**
Age of onset (years)	37.81 (6.83)	38.95 (6.31)	0.04^*^
Median (Min, Max)	34.0 (22.00, 46.00)	39.0 (22.00, 49.00)	
Diagnostic delay (months) Median (Min, Max)	10 (3, 24)	12 (3, 48)	0.20
Site of onset			
Bulbar	2 (22.22%)	9 (20.93%)	0.93
Spinal	7 (77.78%)	34 (79.07%)	
ALSFRS-R total score at baseline	38.78 ± 5.97	37.42± 5.59	0.52
Δ ALSFRS-R	0.58 (0.11,6.00)	0.64 (0.11,4.50)	0.89
Medication use			0.27
Yes	7 (77.78%)	39 (90.70%)	
No	2 (22.22%)	4 (9.30%)	

* *The mean age of onset was slightly younger in the within 1 year group than in the over 1 year group*.

### Linear Regression Analysis Assessing the Association Between Clinical Features and Progression Rate in the Female Cohort

In the univariate analysis, diagnostic delay was highly correlated with the disease progression, with short delay representing rapid progress (*p* = 0.04). After adjustment for confounding factors in the cohort of 52 female patients of childbearing age, we failed to find any difference between the within 1 year group and over 1 year group. And previous pregnancy did not affect disease progression. In the above model, no multicollinearity was found among every variable (Table 3).

**Table 3:** Linear regression analysis assessing the association between clinical features and progression rate in the female cohort.

	**Univariate analysis**		**Multivariate analysis**	
	**OR (95% CI)**	***p*** **-value**	**Adjusted OR** **(95% CI)**	***p*** **-value**
Age at onset	−0.02 (−0.07,0.03)	0.34	−0.06 (−0.13,0.02)	0.15
Age at first pregnancy	−0.01 (−0.10,0.07)	0.75	−0.05 (−0.15,0.04)	0.27
Site of onset				
Spinal onset	Reference		Reference	
Bulbar onset	0.69 (−0.11,1.47)	0.09	0.91 (−0.13,1.96)	0.09
Medication use				
No	Reference		Reference	
Yes	0.15 (−0.89,1.19)	0.77	−0.11 (−1.27,1.05)	0.85
Diagnostic delay	−0.04 (−0.07,0.00)	0.04^*^	−0.03 (−0.07,0.02)	0.19
Previous pregnancy				
Within 1 year	Reference		Reference	
Over 1 year	−0.32 (−1.19,0.55)	0.47	−0.12 (−1.25,0.10)	0.83

* *The diagnostic delay was highly correlated with the disease progression, with short delay representing rapid progress*.

### Description of the Patients' Previous Pregnancy and Fertility

Eight patients were excluded from the analysis, five of whom could not recall when they were first pregnant, and 3 of whom were never pregnant. The mean age of first pregnancy was 25.57 years (± 4.40), with a median age of 25.00 years. The mean age of first pregnancy in the over 1 year group was younger than that in the within 1 year group among the remaining 44 cases. (*p* = 0.01). The number of pregnancies and live births did not differ between the two groups (Table 4).

**Table 4:** Comparison of previous pregnancy and fertility between ALS patients with symptom onset within 1 year and over 1 year after symptom onset.

	**Within 1 year (*N* = 9)**	**Over 1 year (*N* = 43)**	***p*** **-value**
Age of first pregnancy	28.78 (4.77)	24.74 (3.96)	0.01^*^
Median (Min, Max)	30.00 (21.00,34.00)	25.00 (16.00,35.00)	
Number of pregnancies			0.42
0	0	3	
1	5	13	
2	3	11	
3	0	8	
>3	1	7	
Number of live births			0.33
0	2	3	
1	4	17	
2	3	14	
>2	0	8	

* *The mean age of first pregnancy was significantly higher in the within 1 year group than that in the over 1 year group*.

In the within 1 year group, three patients had given birth twice, one (33.33%) had experienced spontaneous abortion, and two (66.67%) chosen induced abortion due to being diagnosed with ALS. In the over 1 year group, two patients reported a history of premature birth. All other pregnancies were full-term. Three patients (33.33%) in the within 1 year group and eight patients (18.60%) in the over 1 year group had Cesarean deliveries. All 80 newborns were healthy. The anesthesia modality was not reported in this study because most patients who underwent Cesarean delivery could not recall this parameter.

The temporal relationship and disease progression of patients in the within 1 year group is summarized in Table 5. Although pregnancy was not associated with accelerated disease progression, patient four reported aggravated weakness in both lower limbs at postnatal day 3.

**Table 5:** The temporal relationship and disease progression in patients in the within 1 year group.

**Patients**	**Age of Onset (years)**	**Time of symptom onset to Pregnancy**	**Δ ALSFRS-R**
Pt#01	32	1 month after miscarriage	0.87
Pt#02	27	12 months before	6.00
Pt#03	37	11 months before	0.35
Pt#04	34	1 month after fertility	0.65
Pt#05	36	During pregnancy	2.75
Pt#06	22	During pregnancy	0.11
Pt#07	34	During pregnancy	0.77
Pt#08	46	5 months after fertility	0.27
Pt#09	38	12 months after fertility	0.58

### Genetic Characteristics

**Figure 1:**
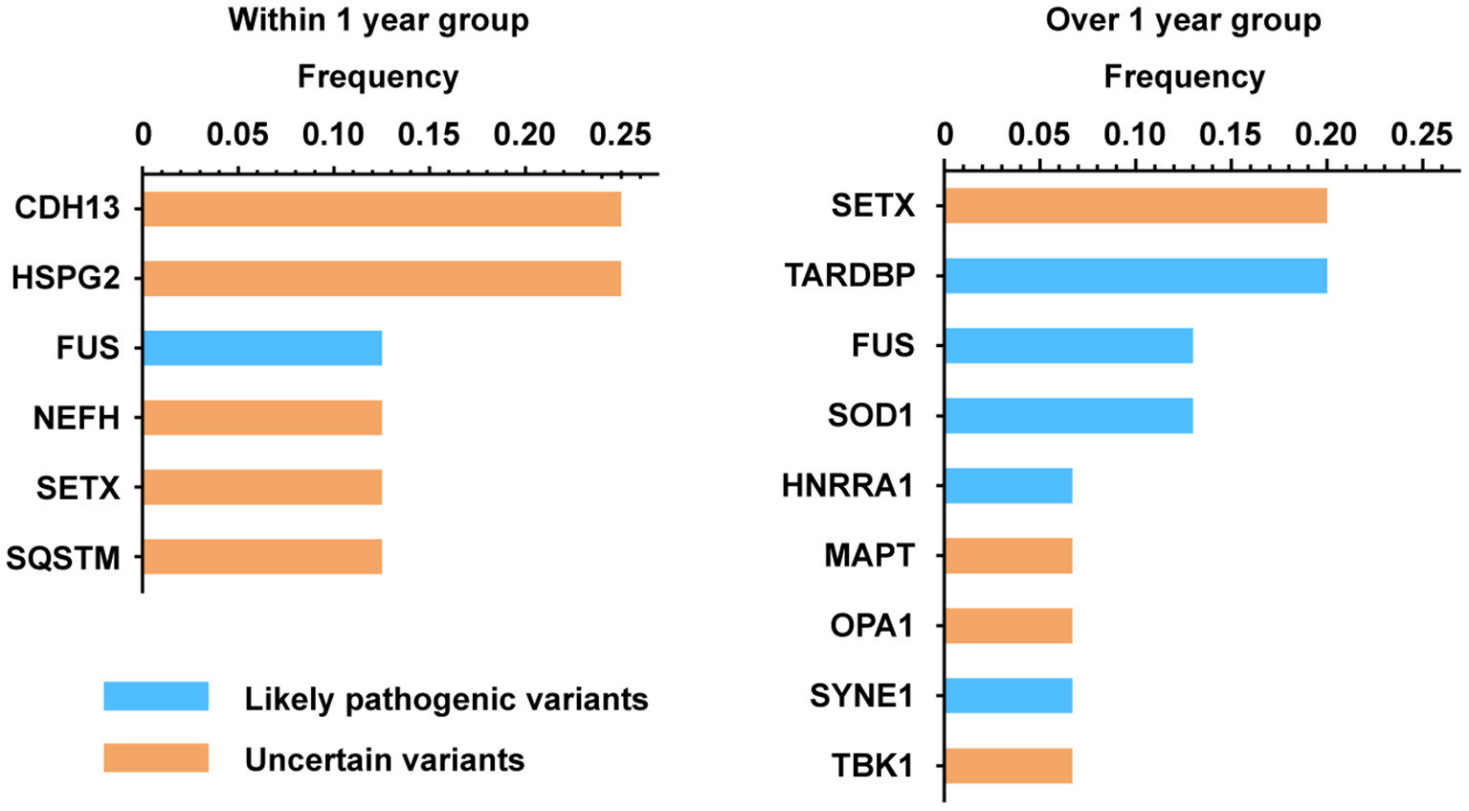
The percentage of patients with ALS at childbearing age carrying gene mutations.

Two of the 52 patients had a family history (3.85%) of ALS. Eighty-four ALS-related genes were analyzed by second-generation sequencing, of which 21 patients (40.38%) were found to carry ALS-related variants, including 18 patients (85.71%) with one variant and 3 patients (14.29%) with two variants. Among the 24 genetic variants, 4 were pathogenic, 6 were likely pathogenic,14 were uncertain (Supplementary Table 2). The proportion with gene mutations in the within 1 year group was 66.67%, higher than that in the over 1 year group (34.88%, *p* <0.01) (Table 6). Interestingly, SETX was the most frequently-mutated gene in this cohort (16.67%), including 4 uncertain mutation (Figure 1), followed by TARDBP and FUS (12.50% each). The mutant frequency of SOD1 was 8.33%. The GGGGCC (G4C2) repeat expansion in C9ORF72 was not observed in our cohort. Due to the small number of patients included in our study, we did not perform further analysis of the association between clinical characteristics and genetic variants.

**Table 6:** The genetic characteristics of patients with ALS at childbearing age.

	**Within 1 year (*N* = 9)**	**Over 1 year (*N* = 43)**	***p*** **-value**
Number of mutations			<0.01^*^
Non-carriers	3	28	
One carrier	3	15	
Two carriers	3	0	

* *The proportion with gene mutations in the within 1 year group was higher than that in the over 1 year group*.

## Discussions

Studies focused on patients with ALS at reproductive age are rare. Fertility status, and clinical and genetic characteristics of these patients have not been reported. Recent studies suggested that changes in estrogen levels may contribute to the onset and progression of several types of ALS (20–24). However, the ability of pregnancy or fertility to exacerbate disease progression has not been evaluated.

Previous studies showed that the annual ALS incidence in females was 1.38 per 100,000 person-years, and patients under 50 years old constituted 39.68% of all patients (2). The mean age at symptom-onset was 53.34 ± 13.55 years in Chinese women. However, the mean age at symptom-onset in patients under 50 years was not reported. In our cohort, the mean age of symptom onset was 37.81 years (± 6.83), with a median age of 38.00 years. In our study, the mean diagnostic delay was 12 (3–48) months, which was significantly shorter than in other reports in China (10, 15, 16). The mean baseline ALSFRS-R total score was 37.65 (± 5.63) (range 24–46), which indicated a relatively milder neurologic deficit.

We found that the mean age of onset in the within 1 year group was slightly younger than that in the over 1 year group (*p*=0.04). This difference might not be due to the disease itself, but by the fact that patients who had given birth for more than one year were likely to be older. We also found that the mean age of first pregnancy in the over 1 year group was lower than that in the within 1 year group. A recent study found that experiencing first pregnancy at or after age 30 was associated with increased risk for being diagnosed with ALS before age 60 (25). Amyotrophic lateral sclerosis is a complex disease caused by both genetic and environmental factors. Since the load on all systems of a woman's body was significantly increased during pregnancy, including motion system, which might mean a greater workload and higher energy metabolic demand for the anterior horn cells, which was a stressful stimulus. Thus, we supposed that younger anterior horn cells might be more resilient to stress for women of childbearing age who were destined to have ALS. In contrast, older anterior horn cells could not adapt to the accelerated pathogenesis of ALS, which triggered the disease onset.

The few studies that evaluated the correlation between pregnancy or fertility and ALS progression have yielded contradictory results. Some case reports indicated rapid progression of ALS during pregnancy (7, 10, 25, 26), while some argued that the patients were stable during the pregnancy and progressed rapidly after delivery (27, 28). Therefore, we compared disease progression between sexes and in different subgroups based on temporal relationship between symptom onset and pregnancy. The median reduction of ALSFRS-R scores did not differ between males and females. Furthermore, there was no difference in reduction of ALSFRS-R scores between the within 1 year and the over 1 year groups. However, one patient reported that weakness in both lower limbs was aggravated at postnatal day 3 in our cohort. We proposed that parturition might represent a trauma that might exacerbate the disease in the short term but not in the long term. The number of patients included in the within 1 year group was small, therefore we were unable to separated patients into subgroups of disease onset before or after conception for further analysis.

In the univariate analysis, diagnostic delay was highly correlated with the disease progression, with short delay representing rapid progress. Similar findings had been found in previous studies that diagnosis delay might be a stronger predictor than the age of onset (29). After adjustment for confounding factors, we failed to find any difference between the within 1 year group and over 1 year group. Considering the small sample size, it was necessary to expand the sample size to further validate our results.

The proportion of gene mutations in the within 1 year group was higher than that in the over 1 year group in our cohort, suggesting that a genetic examination was needed for these patients. The most frequently mutated gene was SETX. Previously, SOD1 was identified as the most frequently-mutated ALS gene in China, followed by TARDBP and FUS (20), while SETX mutation was relatively rare. Mutations in the SETX gene were associated with a rare and autosomal dominant form of juvenile ALS, ALS4, which is characterized by early onset (<25 years) motor neuron dysfunction, slow progression, acute muscle weakness and pyramidal tract signs, and the absence of bulbar and sensory abnormalities (30). The age of onset of our patients with SETX mutations ranged from 27 to 40 years old, which was significantly older than typical patients with ALS4. A study that screened the SETX gene in patients with sporadic ALS in Chinese showed that the mean age symptom onset was 55 years old for patients with SETX mutations, which was similar to the age of onset for the general Chinese population (31). However, all the four SETX mutation in our cohort were uncertain, which still needed further evaluation.

This study was subject to several limitations. The sample size was small, which may have resulted in sample bias. In addition, the possibility of recall bias could not be excluded since some patients had difficulties accurately recollecting the details of their pregnancy when the pregnancy occurred many years in the past. In addition, registration was voluntary, and not every patient diagnosed in our three centers participated in the survey.

## Conclusion

Pregnancy and fertility were not associated with disease progression. Diagnostic delay was likely to be correlated with disease progression in this cohort, and future large-sample analyses were needed to exclude confounding factors to support the conclusion. In addition, SETX might be a gene of concern for ALS patients of childbearing age. Female ALS patients of childbearing age need more attention, and more investigation of ALS should be conducted to explore the relationship between pregnancy factors.

## Data Availability Statement

The raw data supporting the conclusions of this article will be made available by the authors, without undue reservation.

## Ethics Statement

The studies involving human participants were reviewed and approved by the Ethic Committee for Clinical Research and Animal Trials of the Guangdong Provincial Hospital of Chinese Medicine, the First Affiliated Hospital, Sun Yat-sen University and Nanfang Hospital, Southern Medical University. The study approval numbers were H2015-012-02, NFEC-2017-029, and 2021260. The patients/participants provided their written informed consent to participate in this study.

## Author Contributions

BY, BD, YP, and XY designed the study. BY, SH, YZ, XH, JL, and YP collected and analyzed the data. BY, SH, YP, and BD wrote the manuscript. All authors contributed to the article and approved the submitted version.

## Funding

This study was supported by grants from the Scientific research project of Guangdong Provincial Administration of Traditional Chinese Medicine (Grant Nos. 20203002 and 20211188), National Key Research and Development Program of China (2017YFC0907703), Young Scientist Fund of the National Natural Science Foundation of China (82001332), Diagnosis and Treatment of Major Neurological Diseases (2020B1212060017), Guangdong Provincial Clinical Research Center for Neurological Diseases (2020B1111170002), the Southern China International Cooperation Base for Early Intervention and Functional Rehabilitation of Neurological Diseases (2015B050501003 and 2020A0505020004), Guangdong Provincial Engineering Center for Major Neurological Disease Treatment, and Guangdong Provincial Translational Medicine Innovation Platform for Diagnosis and Treatment of Major Neurological Disease.

## Conflict of Interest

The authors declare that the research was conducted in the absence of any commercial or financial relationships that could be construed as a potential conflict of interest.

## Publisher's Note

All claims expressed in this article are solely those of the authors and do not necessarily represent those of their affiliated organizations, or those of the publisher, the editors and the reviewers. Any product that may be evaluated in this article, or claim that may be made by its manufacturer, is not guaranteed or endorsed by the publisher.
