# Supplementary material for: Fertility Does Not Alter Disease Progression in ALS Patients of Childbearing Age: A Three Centers Retrospective Analysis in Southern China
Source: Front Neurol. 2022 Jun 30;13:895321. doi: 10.3389/fneur.2022.895321 (PMC9279572; doi:10.3389/fneur.2022.895321)
Supplement: Supplementary file 1 [file Table_1.DOCX]

**Supplementary Table.1 List of target genes analyzed**

| *ALS2* | *ANG* | *ANXA11* | *APEX1* | *AR* | *ASAH1* | *ATP7A* | *ATXN2* |
| --- | --- | --- | --- | --- | --- | --- | --- |
| *BICD2* | *BSCL2* | *C19orf12* | *C9orf72* | *CDH13* | *CHCHD10* | *CHMP2B* | *CHRM1* |
| *CSF1R* | *DAO* | *DCTN1* | *DNAJB2* | *DYNC1H1* | *ELP3* | *ERBB4* | *FBXO38* |
| *FIG4* | *FUS* | *GRN* | *HFE* | *HNRNPA1* | *HNRNPA2B1* | *HSPB1* | *HSPB3* |
| *HSPB8* | *IGHMBP2* | *KIF5A* | *LMNB1* | *MAPT* | *MATR3* | *NEFH* | *NEK1* |
| *NOP56* | *OPA1* | *OPTN* | *PARK7* | *PFN1* | *PLEKHG5* | *PNPLA6* | *PON1* |
| *PON2* | *PON3* | *PPARGC1A* | *PRPH* | *PRPH2* | *PSEN1* | *REEP1* | *SETX* |
| *SIGMAR1* | *SLC52A1* | *SLC52A2* | *SLC52A3* | *SLC5A7* | *SMN1* | *SMN2* | *SOD1* |
| *SPAST* | *SPG11* | *SPG7* | *SQSTM1* | *SYNE1* | *TAF15* | *TARDBP* | *TBK1* |
| *TREM2* | *TRPM7* | *TRPM7* | *TRPV4* | *TUBA4A* | *UBA1* | *UBQLN2* | *UNC13A* |
| *VAPB* | *VCP* | *VEGFA* | *VPS54* |  |  |  |  |
