# Supplementary material for: Fertility Does Not Alter Disease Progression in ALS Patients of Childbearing Age: A Three Centers Retrospective Analysis in Southern China
Source: Front Neurol. 2022 Jun 30;13:895321. doi: 10.3389/fneur.2022.895321 (PMC9279572; doi:10.3389/fneur.2022.895321)
Supplement: Supplementary file 2 [file Table_2.DOCX]

**Supplementary Table 2. Analysis of the pathogenicity of the mutated genes**

| **gene** | **Transcripts** | **Nucleotides（amino acids）** | **Heterozygous/ Homozygous** | **Highest-MAF** | **GERP++** | **REVEL** | **ACMG scoring** | **ACMG pathogenicity** |
| --- | --- | --- | --- | --- | --- | --- | --- | --- |
| CDH13 | NM_001257 | c.1360G>A （p.V454I） | Heterozygous | 0.0006418 | C(3.84) | B(0.049) | BP4_Moderate | VUS |
| CDH13 | NM_001220492 | c.*2-1C>G splicing | Heterozygous | 0.0054 | NA | NA | BS1 | VUS |
| FUS | NM_004960 | c.1574C>T (p.P525L) | Heterozygous | 0.00000398 | C(2.81) | D(0.735) | PS4+PM1+PM2_Supporting+PM5+PP3 | Pathogenic |
| FUS | NM_004960 | c.1562G>A （p.R521H） | Heterozygous | 0.00000398 | NC(1.83) | LD(0.608) | PS4+PM1+PM2_Supporting+PM5 | Likely pathogenic |
| FUS | NM_004960 | c.1574C>T (p.P525L) | Heterozygous | 0.00000398 | C(2.81) | D(0.735) | PS4+PM1+PM2_Supporting+PM5+PP3 | Pathogenic |
| HNRNPA1 | NM_002136 | c.862C>G (p.P288A) | Heterozygous | NA | C(4.06) | LD(0.605) | PS4+PM1+PM2_Supporting+PM5 | Likely pathogenic |
| HSPG2 | NM_005529 | c.11123G> A (p.R3708Q) | Heterozygous | 0.00001194 | NC(-0.184) | B(0.177) | PM2_Supporting+BP4_Moderate | VUS |
| HSPG2 | NM_005529 | c.5015-3C >T (splicing) | Heterozygous | 0.00112347 | NA | NA | NA | VUS |
| MAPT | NM_001123066 | c.418C> T (p.P140S) | Heterozygous | 0.0082 | C(5.04) | B(0.189) | BP4 | VUS |
| NEFH | NM_021076 | c.172G>T (p.V58L) | Heterozygous | 0.00039936 | C(1.86) | B(0.151) | PM2_Supporting+BS2+BP4_Moderate | Likely benign |
| NEFH | NM_021076 | c.3049G>A (p.A1017T) | Heterozygous | 0.0001 | NC(-11.9) | B(0.224) | PM2_Supporting+BP4 | VUS |
| OPA1 | NM_015560 | c.1363A>G (p.M455V) | Heterozygous | NA | C(5.8) | LD(0.466) | PM2_Supporting | VUS |
| SETX | NM_015046 | c.5403A>C (p.K1801N) | Heterozygous | 0.00032637 | C (3.86) | LD(0.428) | PM2_Supporting | VUS |
| SETX | NM_015046 | c.3992C>T (p.P1331L) | Heterozygous | 0.0059 | C(5.64) | LD(0.437) | BS2 | VUS |
| SETX | NM_015046 | c.754G>A p.A252>T | Heterozygous | NA | C(5.91) | B(0.229) | PM2_Supporting+BP4 | VUS |
| SETX | NM_015046 | c.3992C >T (p.P1331L) | Heterozygous | 0.0059 | C(5.64) | LD(0.437) | BS2 | VUS |
| SOD1 | NM_000454 | c.140A>G (p.H47R) | Heterozygous | NA | C(4.93) | D(0.959) | PS3+PS4+PM1+PM2_Supporting+PM5 | Pathogenic |
| SOD1 | NM_000454 | c.335G>A (p.C112Y) | Heterozygous | NA | C(2.92) | LD(0.653) | PS1+PM1+PM2+PP1_Moderate | Pathogenic |
| SQSTM1 | NM_003900 | c.1104C>A (p.D368E) | Heterozygous | NA | NC (1.38) | B(0.24) | PM2_Supporting+BP4 | VUS |
| SYNE1 | NM_033071 | c.283delG (p.A95fs*19) | Heterozygous | NA | NA | NA | PVS1+PM2_Supporting | Likely pathogenic |
| TARDBP | NM_007375 | c.892G>A (p.G298S) | Heterozygous | 0.00000398 | C(5.81) | LD(0.493) | PS4+PM1+PM2_Supporting+PM5 | Likely pathogenic |
| TARDBP | NM_007375 | c.892G>A (p.G298S) | Heterozygous | 0.00000398 | C(5.81) | LD(0.493) | PS4+PM1+PM2_Supporting+PM5 | Likely pathogenic |
| TARDBP | NM_007375 | c.892G>A (p.G298S) | Heterozygous | 0.00000398 | C(5.81) | LD(0.493) | PS4+PM1+PM2_Supporting+PM5 | Likely pathogenic |
| TBK1 | NM_013254 | c.1674C>G (p.C558W) | Heterozygous | NA | C(2.91) | B(0.057) | PM2_Supporting+BP4_Moderate | VUS |
| UNC13A | NM_001080421 | c.3100C>G (p.L1034V) | Heterozygous | 0.0105 | C (3.52) | B(0.314) | PP2+BS1 | VUS |
